# Facile Synthesis of Water-Soluble Fullerene (C_60_) Nanoparticles via Mussel-Inspired Chemistry as Efficient Antioxidants

**DOI:** 10.3390/nano9121647

**Published:** 2019-11-20

**Authors:** Xiaoyan Zhang, Yihan Ma, Sheng Fu, Aiqing Zhang

**Affiliations:** College of Chemistry and Materials Science, South-Central University for Nationalities, Wuhan 430074, China; zxy517317@163.com (X.Z.); fusheng4715@163.com (S.F.)

**Keywords:** water-soluble fullerene nanoparticles, mussel-inspired chemistry, reactive oxygen species scavenging, cytoprotective effect

## Abstract

Rational design and modification of the all-carbon fullerene cages to meliorate their nature of hydrophobicity is critical for biomedical applications. The outstanding electron affinity of fullerenes enables them to effectively eliminate reactive oxygen species (ROS), the excess of which may lead to health hazards or biological dysfunction. Herein reported is a facile, mild, and green approach to synthesizing the favorable water-soluble C_60_ nanoparticles capable of ROS-scavenging by combining the mussel-inspired chemistry with the Michael addition reaction. Various characterization techniques, including Fourier-transform infrared spectroscopy (FTIR), X-ray photoelectron spectra (XPS), thermogravimetric analysis (TGA), transmission electron cryomicroscopy (Cryo-TEM), and dynamic laser scattering (DLS) were carried out to confirm the satisfactory preparation of the hybrid C_60_-PDA-GSH nanoparticles, which exhibited apparent scavenging capacity of DPPH and hydroxyl radicals in vitro. Additionally, the biocompatible C_60_-PDA-GSH nanoparticles entered into cells and displayed a universal cytoprotective effect against oxidative press induced by H_2_O_2_ in four kinds of human cells at a low concentration of 2 μg/mL. The ease and versatility of the strategy present in this work will not only trigger more fullerene-based materials by the immobilization of diverse functional molecules, but will also extend their possible applications.

## 1. Introduction

Fullerenes, the classic 0-dimensional carbon-based nanomaterials with a closed-cage structure, have spurred wide-spread interest and exploration in various research fields due to their versatility since 1985 [1,2,3]. Constructed of covalently bonded carbon atoms featuring a spherical molecular structure, fullerenes display exceptional properties such as electron deficiency and reactive exteriors, which enable them as functional materials suitable for nanomedicine or drug delivery [3,4,5], cosmetic [6], catalysis [7,8], organic and perovskite solar cells [9,10], etc. [11,12,13]. In particular, thanks to the high electron affinity of fullerenes, they are referred to as “radical sponges” possessing effective capacity to quench reactive oxygen species (ROS). ROS containing hydroxyl radical (•OH), superoxide radical anion (O_2_•^−^), singlet oxygen (^1^O_2_), and hydrogen peroxide (H_2_O_2_), are considered to be mediators of oxidative stress implicating in aging and numerous chronic or acute diseases [14,15]. Therefore, antioxidants are of huge importance for the treatment of ROS-induced disorders and participating in biological processes. It is well established that water-soluble fullerene derivatives are attractive and prominent therapeutic candidates in vitro and in vivo studies aimed at attenuating oxidative stress [16,17,18], since the hydrophobic nature of original fullerenes is the maximum choke point that limits their application in biological systems.

In principle, three common strategies are adopted to overcome the repulsion between fullerenes and water, thereby boosting the solubility of fullerenes in aqueous media: (1) directly producing fullerene colloidal aggregates by solvent extraction with the aid of long-term sonication, (2) solubilizing fullerenes in a non-covalent way by H-bond, π–π stacking, or electrostatic interaction, and (3) chemically modifying fullerenes with functional molecules containing hydrophilic moieties like -OH, -COOH, or -NH_2_, etc. For the nano-C_60_ suspensions, the presence of organic solvent by-products tends to induce toxicity and thus require high-power ultrasound and thorough washing [19,20]. Commonly, solubilizing agents such as γ-CD_x_ [21], polysaccharides [22], or liposomes [23] could encapsulate fullerenes to form host-guest complexes through non-covalent coupling, but the challenge is the lability of a high concentration of hydrophobic guest fullerenes [24]. To date, exohedral functionalization is the most prevailing way to fabricate water-soluble fullerene derivatives. Considerable research efforts have been devoted to polyhydroxylated fullerenes (fullerenols), revealing that their toxicity and biomedical functions are related to the amount of hydroxyl groups modified in different ways on the fullerene carbon cage [25,26,27]. Given the above consequences, it is highly desirable to design and develop a facile and efficient strategy to prepare large-scale bioactive fullerene derivatives, which could get rid of tedious synthetic procedures, harmful additional components, or harsh reaction conditions.

Inspired by the adhesive nature of mussel proteins, polydopamine (PDA) has been served as the pioneering coating material for the design and fabrication of a variety of multifunctional materials [28,29,30]. PDA is capable of adhering to virtually any type of material without regard to its composition, size and morphology, while the entire process just involves simply mixing the substrate with dopamine in a weakly basic aqueous solution for a suitable period of time. Particularly, apart from its powerful binding affinity characteristics, the coating PDA also offers active intermediate platforms for the subsequent construction of versatile material [31,32,33]. Hence, compounds bearing nucleophilic groups like amines and thiols could be covalently immobilized onto PDA-decorated material by the Michael addition reaction or Schiff base reactions, so that diverse functional molecules could be introduced to achieve the promising and exciting applications in the fields of chemistry, material and biology [34,35]. So far, a series of work focusing on PDA-modified carbon nanomaterials has been reported and has aroused more interest and research [36,37]. The Wei and Zhang groups have been dedicated to take advantage of PDA coatings as well as plentiful water-soluble polymers (e.g., PAA, polyPEGMA) to modify carbon nanotubes (CNT), graphene oxide (GO), or nanodiamonds, thus fabricating desirable carbon-based nanomaterials with favorable dispersibility and biocompatibility for potential biomedical applications [38,39,40,41]. In addition, Liu et al. have confirmed that GO-PDA-PEI nanocomposites could act as preeminent adsorbents to capture a large amount of hexavalent uranium from wastewater [42]. Xi et al. also have utilized this strategy to prepare CNT-PDA embedded SPEEK membranes for highly efficient and durable vanadium flow batteries [43]. Nevertheless, there has been little research on fullerene modified by PDA through the above green synthesis. It is extremely worthwhile to combine PDA that possesses biomimetic coatings and nanoplatforms with fullerene for extending the applications of fullerene-based materials in various areas of science, especially in biomedicine.

**Scheme 1:**
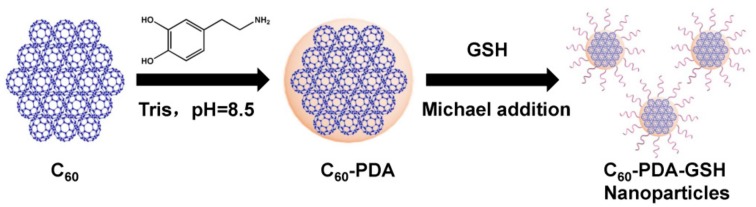
Schematic representation of the preparation of C_60_-PDA-GSH nanoparticles via the combination of mussel-inspired chemistry and the Michael addition reaction.

Herein, we develop a facile and green approach to produce the water-soluble C_60_-PDA-GSH nanoparticles via a two-step method and evaluate their antioxidant performance for the first time. As illustrated in Scheme 1, C_60_ was first decorated by the spontaneous self-polymerization of dopamine to form the C_60_-PDA hybrids, and then the thiol-containing endogenous and bioactive oligopeptide GSH was covalently immobilized onto the PDA-modified C_60_ through the Michael addition reaction. The physical properties and chemical composition of the obtained C_60_-PDA-GSH nanoparticles were investigated by the means of diverse characterization techniques. Notably, the C_60_-PDA-GSH nanoparticles displayed a great capacity for scavenging free radicals, thus exhibiting t cytoprotective effects against oxidative stress in four common kinds of human cells. Moreover, the above synthetic antioxidants were biocompatible without latent cytotoxicity, and the dosage for effective protection was relatively low, which may be beneficial for the treatment of ROS-induced disorders or cosmetics applications.

## 2. Experimental Sections

### 2.1. Materials and Instrumentation

Pristine C_60_ was provided by the Institute of Chemistry, Chinese Academy of Sciences (Beijing, China) with the purity of 99%. Tris-(hydroxymethyl) aminomethane (Tris), dopamine hydrochloride, 2,2-Di(4-tert-octylphenyl)-1-picrylhydrazyl (DPPH), 5,5-Dimethyl-1-pyrroline N-oxide (DMPO) and reduced glutathione (GSH) were purchased from Aladdin Reagent Inc. (Shanghai, China). FITC-Acp-Lys-OH was obtained from GL Biochem Ltd. (Shanghai, China). Hydrogen peroxide solution (H_2_O_2_) was acquired from Sinopharm Chemical Reagents Co. (Beijing, China). Milli-Q (Millipore, Billerica, MA, USA) purified water (18.2 MΩ) was served for preparing all the solutions, and other common reagents were commercially available and served without further purification.

Transmission electron cryomicroscopy (Cryo-TEM) images were examined on a TEM (JEM-2010, JEOL, Tokyo, Japan) at 94 K and recorded by a CCD camera (Gantega, Olympus Soft Imaging Solutions, Munster, Germany). The Cryo-TEM specimens were made with a Leica EM GP immersion freezer (Wetzlar, Germany) by first dropping 3 μL nanoparticles water suspension onto a glow-discharged carbon coated holey grid (GIG). Then, the above drop was blotted dry with filter paper to obtain a thin liquid layer (TEM grid). Finally, the grid was rapidly submerged into the liquid ethane, which was cooled by liquid nitrogen and the vitrified samples were transferred onto the low temperature Sample holder (626, Gatan, CA, USA). The hydrodynamic diameter and Zeta Potential of samples in Milli-Q water at pH 7.0 were determined thrice on a Zetasizer Nano ZSP instrument (Malvern, UK). Fourier-transform infrared spectra (FTIR) studies of pristine C_60_, C_60_-PDA, and C_60_-PDA-GSH were carried out on a Thermo Nexus470 spectrometer (Waltham, MA, USA). Raman spectra were carried out on a DXR2xi Raman Imaging Microscope (Waltham, MA, USA) with a 532 nm excitation wavelength. X-ray photoelectron spectra (XPS) were performed on a VG ESCALab220i-XL spectrometer (East Grinstead, UK) with an Al Ka X-ray source (1486.6 eV), and the binding energy data were calibrated for C1s at 284.6 eV. Thermogravimetric analysis (TGA) was carried out on a TA instrument Shimadzu DTG-60H (Kyoto, Japan), under N _2_ atmosphere at a heating rate of 10 °C/min. The UV-vis absorbance of DPPH with samples at 516 nm was measured on a Shimadzu UV-2550 spectrophotometer (Kyoto, Japan). Electron spin resonance (ESR) spectroscopy was recorded on a JEOL JEF FA200 EPR (Tokyo, Japan) spectrometer at ambient temperature.

### 2.2. Preparation of C_60_-PDA-GSH

C_60_-PDA was prepared according to our reported method [44]. For the synthesis of C_60_-PDA-GSH nanoparticles, 50 mg C_60_-PDA and 100 mg reduced glutathione were dispersed into 20 mL NaOH aqueous solution (0.1 M) and stirred overnight at room temperature to give an orange aqueous solution. These as-synthesized C_60_-PDA-GSH nanoparticles were purified through a 220 nm membrane filter and dialyzed by a porous cellulose bag (MWCO = 3500 Da) for 3 days to remove free GSH and NaOH. Finally, the harvested products (C_60_-PDA-GSH) were collected by freeze drying for further characterization.

### 2.3. DPPH Scavenging Activity of C_60_-PDA-GSH

As a stable nitrogen-centered free radical, DPPH radical was employed to evaluate the radical-scavenging ability of C_60_-PDA-GSH nanoparticles based on the attenuation of the UV-vis absorbance at 516 nm. In detail, 0.1 mM of DPPH in ethanol was freshly prepared before measurement. A range of samples dispersed in water at different concentrations was incubated with the same volume DPPH/ethanol solution for 30 min in the darkness. Then, the UV absorbance of different mixtures at 520 nm was recorded, respectively. In addition, the radical scavenging activity of samples against DPPH was calculated according to the equation: *I* = [1 − (A_i_ − A_j_)/A_c_] × 100%, where A_i_ is the absorbance of sample mixed with DPPH, A_j_ is the absorbance of pure sample, and A_c_ is the absorbance of pure DPPH solution in the absence of samples [45].

### 2.4. Scavenging Effects of C_60_-PDA-GSH on Hydroxyl Radicals

Interception of •OH yielded by ultraviolet (UV) irradiation of H_2_O_2_ was determined by the ESR spin-trapping method [46]. In addition, 100 µL DMPO (100 mM) as the spin-trapping reagent, 50 µL H_2_O_2_ (1 M), and 50 µL C_60_-PDA-GSH (150 and 200 µg/mL) were mixed together, respectively. Then, the above solution was irradiated by a UV lamp (500 W) for 4 min, and conducted on ESR measurements immediately. DMPO captures hydroxyl radicals to form DMPO-OH, which was detected in the darkness with a cumulative time of 1 min. The quenching ability of C_60_-PDA-GSH toward hydroxyl radical was evaluated by comparison with the matched group.

### 2.5. Cell Culture and Treatment

Human epidermal keratinocytes (HEK-a), human umbilical vein endothelial cells (HUVEC), human microglia (HM), and normal liver cells (L-02) cells were acquired from the Nanjing Cobioer Biotechnology Institute, China. The complete medium of different cells was prepared as follows. The medium used for both HEK-A and HUVEC cells was high glucose Dulbecco’s minimal essential medium (DMEM) medium (glucose concentration 4.5 g/L) complemented with 10% fetal bovine serum (FBS) and 1% penicillin-streptomycin double-antibody solution. As for L-02 cells, RPMI 1640 basic medium (L-Glutamine and 25 mM HEPES, Gibco, CA, USA) was applied, while low glucose DMEM medium (glucose concentration 1.0 g/L) was used for HM cells. All of the cells were cultured in a cell box under the culture conditions of 37 °C, 5% carbon dioxide.

### 2.6. Cytotoxicity Test Using HEK-a Cells

To assess the cytotoxicity of C_60_-PDA-GSH nanoparticles in the darkness, HEK-a cells were selected as the cell model. The cell was cultured in a 96-well plate with a density of 1 × 10^4^/well at 37 °C for 24 h and then incubated with different concentrations of the samples (25, 50, 100, 150, and 200 μg/mL) for 24 h in the darkness. Afterwards, the Cell Counting Kit-8 (CCK-8; DOJINDO, Kumamoto, Japan) assay was performed to detect the cell viability. The absorbance was measured at 450 nm by a microplate reader (Infinite M1000Pro Tecan i-control, Mannedorf, Switzerland) to calculate the cell viability.

For the cytotoxicity examination under visible light, the HEK-a cells were cultured in a 96-well plate at 37 °C for 24 h, and then incubated with the samples (150, 200 μg/mL) for 3 h. Subsequently, the cell was exposed to white light (20 mW) for 10 min, and then incubated with free culture medium for 24 h in the darkness. In the end, the CCK-8 method was employed to evaluate the cell viability.

### 2.7. Protection of Cells against Oxidative Stress

To investigate how C_60_-PDA-GSH nanoparticles protect various cells from H_2_O_2_-induced oxidative stress, a series of samples of different concentrations in phosphate buffered saline (PBS) were added to the HEK-a, HUVEC, HM and L-02 cells and cultured for 3 h separately. Then, it was treated with 1 mM H_2_O_2_ for hours. Thereafter, the medium was removed, and the cells were cultured for 24 h with free medium to measure the cell viability by the CCK-8 assay.

### 2.8. Fluorescence Costaining and Imaging

To investigate the cell internalization of C_60_-PDA-GSH nanoparticles by cells uptake, C_60_-PDA-GSH was labeled with a fluorescent dye (FITC-Acp-Lys-OH), also through the Michael addition reaction between a thiol group and PDA. Then, HEK-a cells were stained with dye Hoechst 33342 (Solarbio, Beijing, China), DiI (Beyotime, Shanghai, China), and MitoTracker deep red (Invitrogen, CA, USA) separately to determine the intracellular position of fluorescent C_60_-PDA-GSH.

The JC-1 staining assay was carried out to differentiate the mitochondrial membrane potential, which is a landmark event in the early stages of apoptosis. For fluorescence observations, before treatment with H_2_O_2_ for 1 h, HEK-a cells were first incubated with the samples for 3 h. Subsequently, the cells were stained with JC-1 dye for 20 min at 37 °C in the darkness and then washed with buffer. Images of fluorescence were obtained by a confocal laser scanning microscope (Olympus FV 1000-IX81, Tokyo, Japan). A laser with a wavelength of 488 nm was selected to excite JC-1 monomer (representing depolarized mitochondrial membrane potential) and detected at 500 to 535 nm, while laser of 559 nm was used for JC-1 polymer (representing polar mitochondrial membrane potential) and recorded from 600 to 635 nm.

## 3. Results and Discussion

### 3.1. Characterizations of C_60_-PDA-GSH Nanoparticles

**Figure 1:**
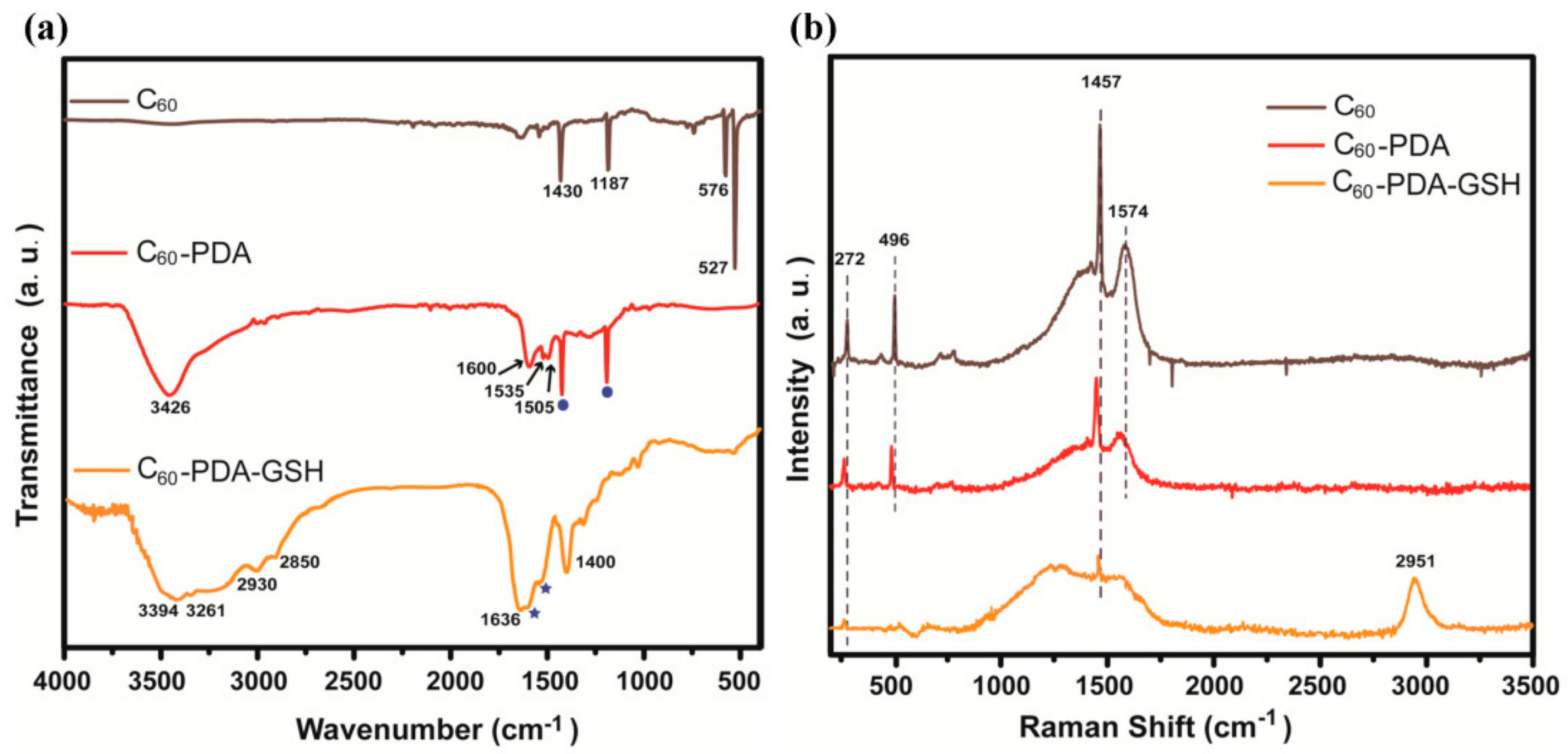
FTIR (**a**) and Raman spectra (**b**) of C_60_, C_60_-PDA and C_60_-PDA-GSH. The solid circles and asterisks label the signals from C_60_ and PDA, respectively.

Herein, we synthetically developed a gentle and viable procedure for the well water-soluble C_60_-PDA-GSH nanoparticles through mussel-inspired chemistry. In order to demonstrate the successful preparation of the expected product, FTIR analysis was first performed during the preparation process_._ As depicted in Figure 1a, four characteristic signals of pristine C_60_ powders appeared at 1430, 1187, 576, and 527 cm^−1^, which should be recognized as the three-fold degenerate first-order dipole active *T*_1u_ modes, and other peaks could be ascribed to the solvent peaks of toluene. It is apparent that, after chemical modification, several new absorption peaks emerged between 1600 and 1450 cm^−1^ due to the skeleton stretching vibration of the polycyclic aromatic ring, and the broad band close to 3426 cm^−1^ arising from O-H and N-H stretch further proved that C_60_ had been wrapped by PDA successfully via mussel-inspired chemistry. Compared with C_60_ and C_60_-PDA, the intensive absorption peak of amide I located at 1636 cm^−1^ and amide III at 1400 cm^−1^ could be identified as the stretching vibration of C=O and C-N bonds, respectively, indicating the presence of GSH. Meanwhile, two characteristic absorption peaks at 2930 and 2850 cm^−1^ corresponding to the stretching mode of methylene as well as the broader band around 3380 cm^−1^ could also be observed in the spectrum of C_60_-PDA-GSH. These data further confirmed the successful modification of the pristine C_60_ with PDA and GSH. Raman spectroscopy complementary to IR investigation was also carried out to verify the modification of C_60__._ As shown in Figure 1b, two Hg modes at 272 cm^−1^ and 1574 cm^−1^, the Ag-breathing mode at 496 cm^−1^ as well as the Ag-pentagonal pinch mode at 1457 cm^−1^ were the four characteristic peaks of C_60_. In the case of C_60_-PDA, no new peaks were observed, which was consistent with the results of CNT-PDA [47] and GO-PDA [36]. Nonetheless, these four peaks were slightly shifted downwards, presumably due to the vibronic coupling mechanism of electron transfer between C_60_ and PDA [48,49]. Moreover, for C_60_-PDA-GSH, the original vibrational signals of C_60_ near 272 cm^−1^ and 1457 cm^−1^ still remained. A broad peak band without fine structure between 750 and 1750 cm^−1^ appeared, probably due to the complicated interaction between PDA and GSH, since PDA didn’t have an accurate structure [28]. In addition, an apparent peak emerged at 2951 cm^−1^, which could be assigned to the stretching vibration of C-H bonds, suggesting the presence of GSH. Therefore, the above results further consolidated the successful attachment of GSH and PDA on the pristine C_60_.

**Figure 2:**
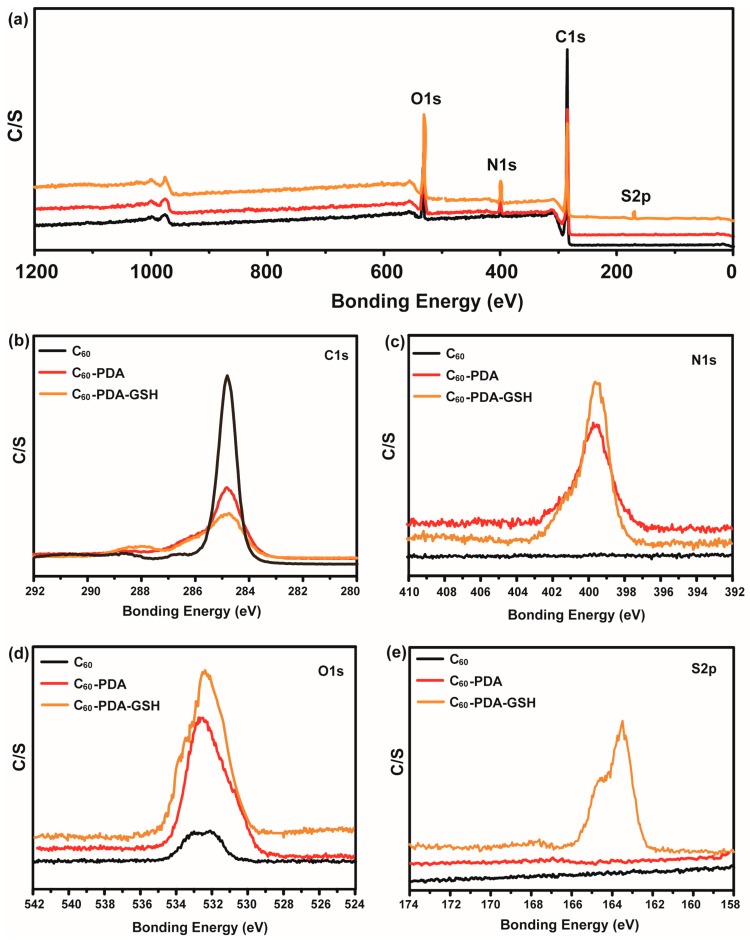
XPS spectra of C_60_, C_60_-PDA and C_60_-PDA-GSH (**a**) survey scan of the spectral region from 0 to 1200 eV, (**b**) C1s region, (**c**) N1s region, (**d**) O1s region, and (**e**) S2p region.

Further evidence of valid functionalization could be obtained by X-ray photoelectron spectroscopy (XPS) experiments to study the detailed chemical composition and state of the prepared species. As shown in Figure 2a, the main elements of carbon (C), nitrogen (N), oxygen (O), and sulfur (S) were determined in a series of C_60_ samples with a survey scan range of 1200 to 0 eV. In contrast to virgin C_60_, both C_60_-PDA and C_60_-PDA-GSH exhibited characteristic peaks of nitrogen around 400 eV, manifesting the attachment of PDA to C_60_. Significantly, the existence of sulfur element in the C_60_-PDA-GSH could be attributed to the sulfydryl of GSH. In detail, Figure 2b–e displayed the high resolution XPS spectra of C1s, O1s, N1s and S2p, respectively. A symmetrical peak centered at 284.9 eV was observed in the C1s spectral region of C_60_, which was related to the C-C and C=C bonds of the non-functionalized sp^2^-hybridized carbon atoms in the fullerene skeleton ring. As for C_60_-PDA, additional fitting peaks (287.5 and 286.1 eV) for C1s indicated disparate chemical environments i.e., C=O and C-N, respectively (Figure 2b and Figure S1a). In addition, the peak of N1s of C_60_-PDA located at 399.9 eV could be assigned to the nitrogen heterocyclic structure of the adhered PDA, and the enhanced intensity of the O1s peak between 530 and 535 eV could also be attached to the catechol groups of PDA. After modification with GSH, the intensity of the N1s as well as the O1s peak slightly increased compared with the spectra of C_60_ and C_60_-PDA, while the C1s peaks were relatively attenuated. Most importantly, the binding energy peak of S2p between 165.8 and 162.2 eV was exclusively observed in the spectra of C_60_-PDA-GSH. More specifically, due to their spin–orbit couplings, the S2p profile could be curve-fitted into two characteristic sulfur 2p_3/2_ and 2p_1/2_ peaks at 163.5 and 164.7 eV, respectively (Figure S1c), in line with the reported C-S covalent bonds in an aromatic sulfide compound [50], strongly corroborating that thiol-containing GSH could be further linked to the C_60_-PDA. In addition, the element compositions of the C_60_ samples according to the XPS spectra were also listed in Table 1. The original C_60_ composed of a large amount of C atoms (97.50%) and a small fraction of O atoms (2.50%), revealing somewhat oxidation but no other impurities. After coated by PDA, the atom contents of C and O in C_60_-PDA were altered to 77.41% and 16.11%, respectively. Notably, the presence of N component (6.48%) derived from the pyrrolic-N units in PDA confirmed that C_60_ could be decorated via mussel-inspired chemistry. When C_60_-PDA-GSH was involved, four elements C (66.51%), O (22.14%), N (9.08%) and S (2.27%) were identified. It is worth noting that the exclusive appearance of S component as well as the enhancement of the N and O contents could be ascribed to the incorporation of GSH. Consequently, all of the above analysis demonstrated the strategy on the basis of the combination of mussel-inspired chemistry and Michael addition reaction could be an alternative, facile, and versatile way for producing C_60_-PDA-GSH nanoparticles.

**Figure 3:**
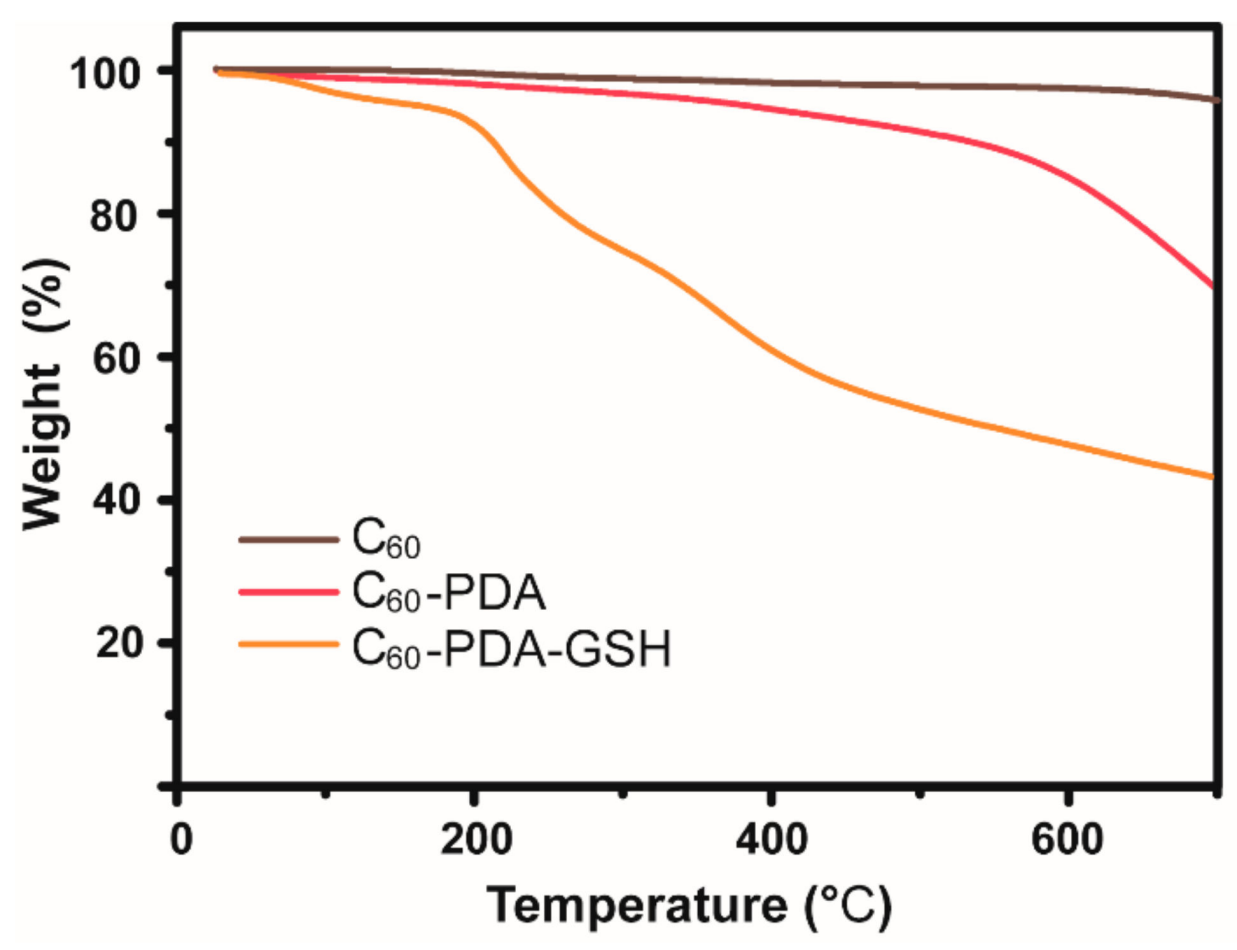
TGA curves of C_60_, C_60_-PDA and C_60_-PDA-GSH, respectively.

Furthermore, we studied the physicochemical properties of the samples by thermogravimetric analysis (TGA) under a nitrogen atmosphere, and the pyrolysis curves for C_60_, C_60_-PDA and C_60_-PDA-GSH were illustrated and compared in Figure 3. Pristine C_60_ displayed overwhelmingly high thermal-stability with a mass residual ratio of 95.9 wt% when the temperature was continuously raised to 700 °C. After surface modification of PDA, no significant thermal decomposition was observed in view of the complicated composition of PDA, and the mass residue of C_60_-PDA was reduced to 69.4 wt%. The increased weight loss could be ascribed to the combustion of the adhesive PDA, since the pure PDA was reported to completely decompose at up to 600 °C [51]. When GSH was introduced into C_60_-PDA by the aid of Michael addition reaction, the residue in C_60_-PDA-GSH was much less in comparison with C_60_ and C_60_-PDA. Accordingly, the apparent weight loss of C_60_-PDA-GSH started at about 200 °C and eventually increased to 57.1 wt%, due to the lability of the oligopeptide GSH, which has a decomposition temperature of 193 °C. Thus, we could conclude that C_60_ was indeed functionalized by PDA and GSH, on account of the differentiation in the above pyrolysis process.

**Figure 4:**
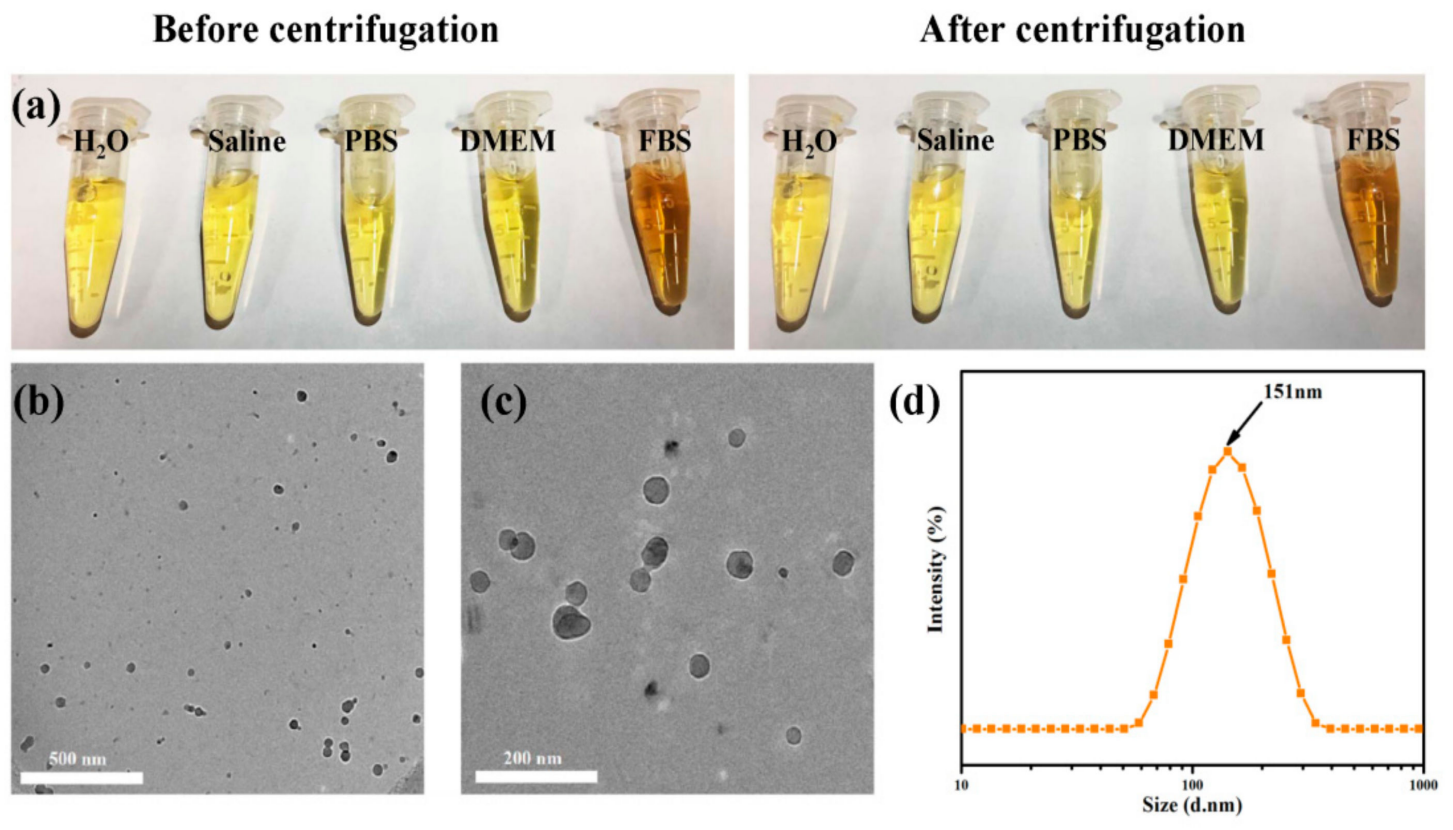
(**a**) photographs of C_60_-PDA-GSH in water, saline, PBS, and DMEM and FBS before and after centrifugation at 10,000 rpm for 10 min. (**b**,**c**) representative cryo-TEM images of C_60_-PDA-GSH with different magnification; (**d**) hydrodynamic size distribution of C_60_-PDA-GSH in water.

The key to implementing the potential application of fullerenes in clinical practice is to promote their solubility in aqueous media. Therefore, the outstanding solubility of the obtained C_60_-PDA-GSH in water as well as varied physiological media was displayed in Figure 4a. Even after centrifugation at 10,000 rpm for 10 min, the transparent solution remained stable without visible sediment and could pass through the 220 nm membrane filter smoothly, while C_60_ and C_60_-PDA totally precipitated under the same condition. Such superior stability of C_60_-PDA-GSH in physiological solutions could facilitate its biomedical function. Subsequently, the analysis by Cryo-TEM of C_60_-PDA-GSH identified that it had an irregular spherical structure with a diameter between 30 and 60 nm (Figure 4b,c). Moreover, we inferred from the results of dynamic laser scattering (DLS) that it was prone to self-associate and form monodisperse nanoparticles in aqueous solution, owing to the intermolecular hydrogen bonds interactions (Figure 4d). The resultant C_60_-PDA-GSH in water had a uniform hydrodynamic size of 151 nm with the dispersibility index (DPI) of 0.205, which was suitable for cellular uptake. In addition, the negatively-charged value of ζ potential (−24.5 eV) also boosted its stability in aqueous media (Figure S2). Thus, the solubility of C_60_-PDA-GSH nanoparticles has been greatly improved on account of the hydrophilic oligopeptide GSH, making them a latent candidate in the field of biomedicine.

### 3.2. Scavenging Free Radicals Activity of C_60_-PDA-GSH Nanoparticles

**Figure 5:**
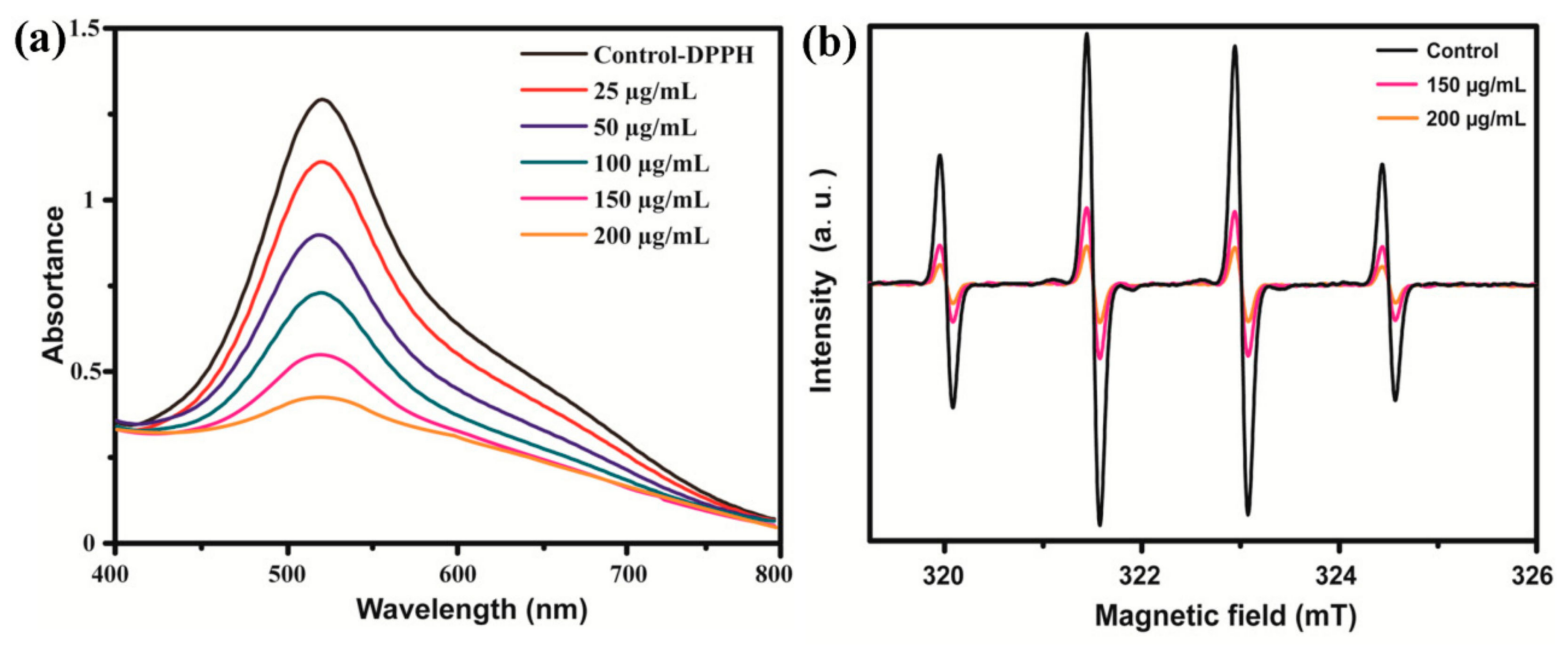
(**a**) DPPH radical scavenging activity of C_60_-PDA-GSH nanoparticles; (**b**) X-band EPR spectra of the DMPO-OH with different doses of C_60_-PDA-GSH nanoparticles.

Afterwards, we first performed a widely used model for removing stable DPPH radicals to assess the antioxidant capacity of C_60_-PDA-GSH nanoparticles in vitro. The DPPH assay is based on the single electron pairing of the DPPH radicals in the presence of antioxidants, converting the original purple DPPH solution to the yellow diphenylpricrylhydrazine solution. The radical-scavenging ability of C_60_-PDA-GSH was investigated by recording the decrease in absorbance at 520 nm (*A*_560_), which is directly related to content of DPPH free radicals. As presented in Figure 5a, the intensity of *A*_560_ regularly declined associated with the increased addition levels, demonstrating that the tested C_60_-PDA-GSH nanoparticles expressed the capacity to scavenge DPPH free radicals in a dose-dependent trend and could eliminate up to 92.7% DPPH radicals with a mass concentration of 200 μg/mL. Simultaneously, similar results could also be acquired by evaluating the capability of C_60_-PDA-GSH to intercept hydroxyl radicals, which was performed on the ESR spectroscopy (Figure 5b). The •OH generated by the ultraviolet excitation of H_2_O_2_ was immediately trapped by DMPO to produce a stable DMPO-OH adduct, exhibiting a characteristic four-line ESR signal. After treatment with C_60_-PDA-GSH nanoparticles, the EPR profiles from the spin adduct DMPO-OH were considerably attenuated, indicating that most of the •OH was actually quenched by the sample. Considering the reduction in signal intensity, approximately 86.2% of the hydroxyl radical was eliminated at a dose level of 200 μg/mL. Therefore, such advantageous free radical affinity and potent quenching activities of C_60_-PDA-GSH nanoparticles in vitro enable them a favorable antioxidative agent for preventing oxidative stress, so the corresponding behavior at cellular level was discussed below.

### 3.3. Celluar Performance of C_60_-PDA-GSH Nanoparticles

**Figure 6:**
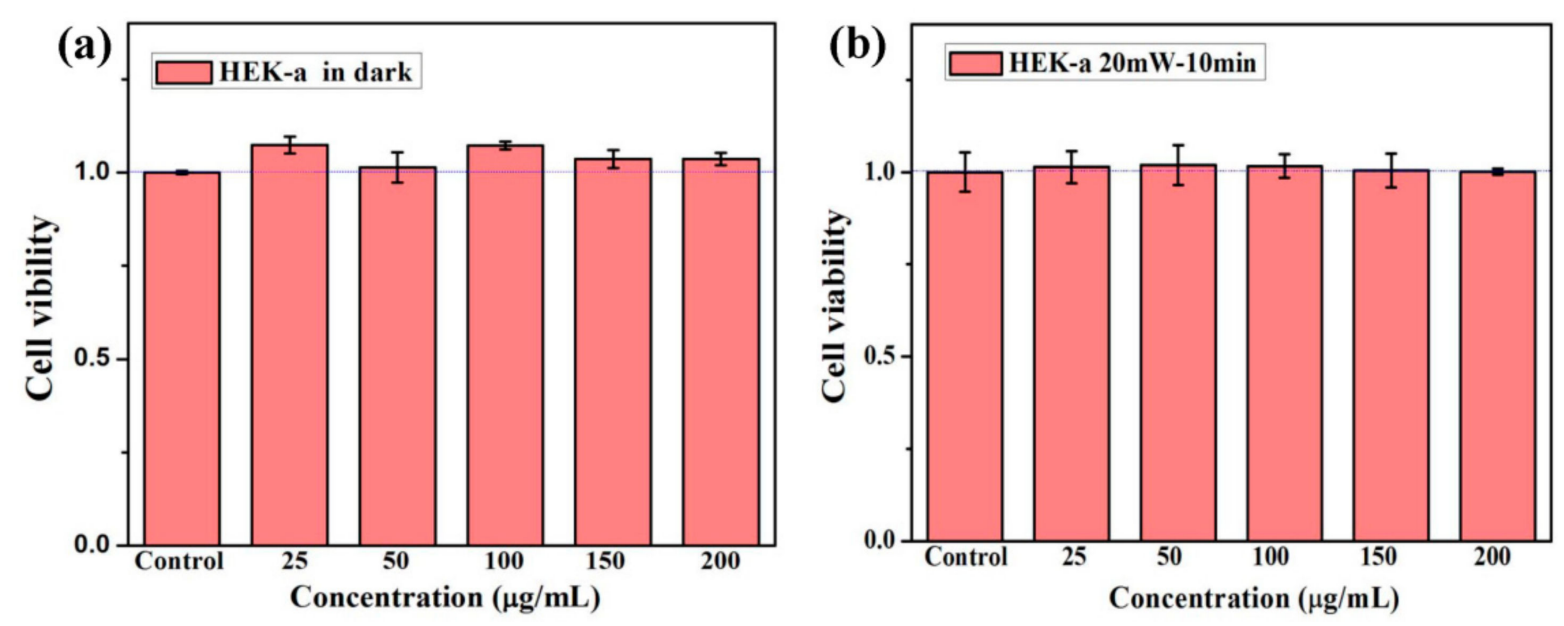
Cell viability of HEK-a cells incubated with different doses of C_60_-PDA-GSH nanoparticles (**a**) in the darkness for 24 h and (**b**) exposed to white light (20 mW/cm^2^) for 30 min and then for 24 h in the darkness. The control groups were the cells treated with PBS.

We systematically investigated the biocompatibility of C_60_-PDA-GSH nanoparticles by examining the latent cytotoxicity of the samples towards HEK-a cells in the presence and absence of light, respectively. Figure 6a plotted the viability of HEK-a cells incubated with C_60_-PDA-GSH nanoparticles at various dosages ranging from 25 to 200 μg/mL in the darkness for 24 h. It is worth mentioning that, even at levels as high as 200 μg/mL, all cells’ viability values increased to some extent, indicating that C_60_-PDA-GSH nanoparticles were not cytotoxic and might promote the cell proliferation in the darkness. Furthermore, when the cells incubated with the samples were exposed to white light irradiation (20 mW/cm^2^), almost all of the identical cell viability was observed compared to the control group (Figure 6b), suggesting that C_60_-PDA-GSH nanoparticles were more inclined to be radical scavengers rather than ROS producers. In summary, the C_60_-PDA-GSH nanoparticles exhibited negligible cytotoxicity with or without irradiation, which is a prerequisite for applications in cosmetics and biomedicine. Given the intrinsic nature of C_60_ as well as the biosafety of PDA and GSH, the profitable biocompatibility of C_60_-PDA-GSH nanoparticles could be expected.

**Figure 7:**
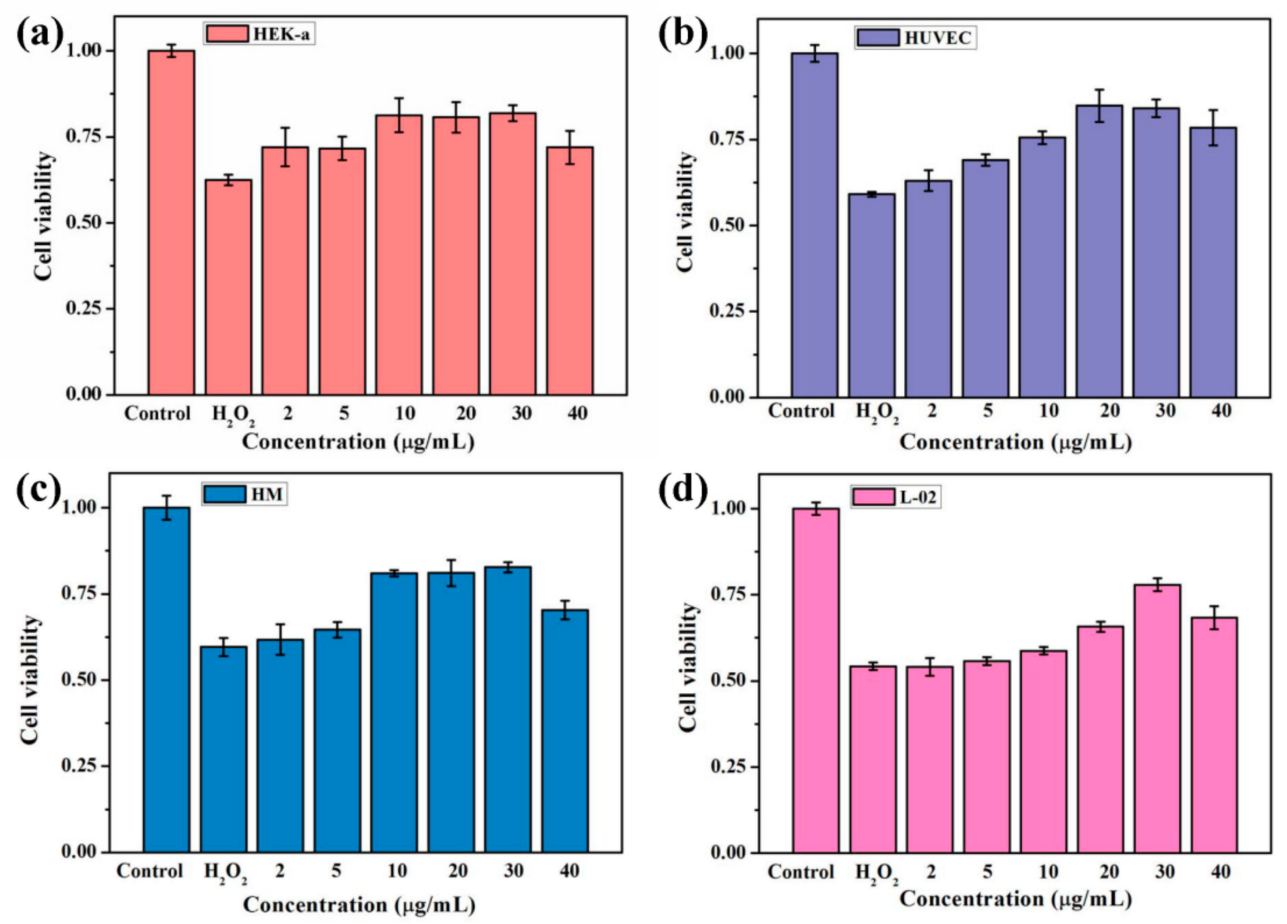
Cell viability of (**a**) HEK-a, (**b**) HUVEC, (**c**) HM and (**d**) L-02 cells that were pre-treated with different concentrations of C_60_-PDA-GSH nanoparticles separately for 3 h before exposure to 1 mM H_2_O_2_ for 1 h. The control groups were the cells treated only with PBS.

To investigate the universal protective effects of C_60_-PDA-GSH nanoparticles against oxidative press induced by H_2_O_2_, we selected HEK-a, HUVEC, HM, and L-02 cells as cultured cells. As illustrated in Figure 7, the viability of all cells decreased with apoptosis when exposed to 1 mM H_2_O_2_. Nevertheless, pretreatment of the four kinds of cells with different concentrations of C_60_-PDA-GSH nanoparticles at 2 to 40 μg/mL remarkably prevented the cell from apoptosis. Compared to the blank group, the survival rate of HEK-a cells pre-treated with only 2 μg/mL prior 3 h to the addition of H_2_O_2_ statistically was boosted to 72%, while the H_2_O_2_-treated cells declined to 52%. In addition, C_60_-PDA-GSH nanoparticles presented a concentration-dependent protection against cytotoxicity, very consistent with the experimental results of quenching free radicals. At a dose of 30 μg/mL, it displayed the best performance, alleviating the H_2_O_2_-induced cell apoptosis to 18%. The relatively reduced cell viability at 40 μg/mL might be attributed to the changes of osmotic pressure in the cell culture environment, but it was yet higher than the group treated with H_2_O_2_ only. Particularly, the identical protective role of C_60_-PDA-GSH nanoparticles against cellular oxidative injury was also observed on other three cells. The H_2_O_2_-induced death of HUVEC, HM and L-02 cells was still inhibited by the pretreatment of C_60_-PDA-GSH nanoparticles at a concentration as low as 2 μg/mL. Especially, C_60_-PDA-GSH nanoparticles at a concentration of 30 μg/mL possessed the highest protective effect, resulting in the cell viability being 84%, 83%, and 78%, respectively. Taking into account the above results, we could infer that C_60_-PDA-GSH nanoparticles may be served as latent antioxidative agents, attributed to their comparative low available concentration and favorable biocompatibility.

**Figure 8:**
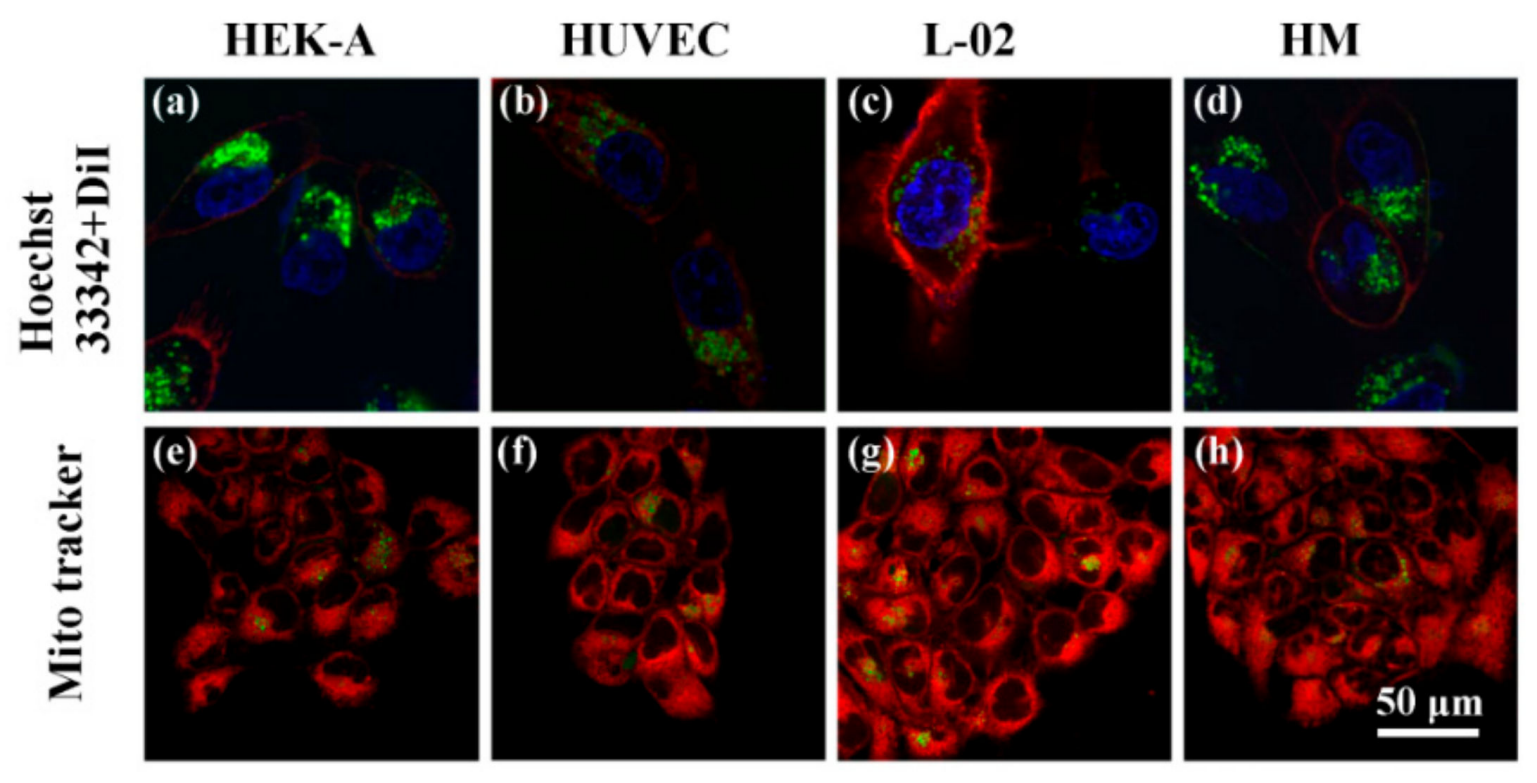
Intracellular localizations of fluorescein-labeled C_60_-PDA-GSH nanoparticles in (**a**,**e**) HEK-a, (**b**,**f**) HUVEC, (**c**,**g**) HM, and (**d**,**h**) L-02 cells, separately. After 3 h of co-incubation, cells were fixed and analyzed by confocal microscopy. Green fluorescence represents C_60_-PDA-GSH nanoparticles, red fluorescence displays DiI-stained cell membrane, blue fluorescence indicates Hoechst 33342-stained nuclei, and deep red fluorescence illustrates MitoTracker-stained mitochondria.

To investigate whether C_60_-PDA-GSH nanoparticles are efficiently internalized to act as intracellular antioxidants, we introduced a thiol-containing green fluorescent dye (FITC-Acp-Lys-OH) which was conjugate with C_60_-PDA-GSH by the routine Michael addition reaction. Hence, after 3 h of co-incubation, the cellular uptake of the fluorescent-labeled C_60_-PDA-GSH nanoparticles in the above four kinds of human cells were monitored by tracking the fluorescent signal, respectively. As depicted in Figure 8a–d, the green intracellular fluorescence of C_60_-PDA-GSH nanoparticles was observed between the areas of cytomembrane stained with DiI (red fluorescence) and nucleus stained with Hoechst 33342 (blue fluorescence) in the HEK-a, HUVEC, HM, and L-02 cells, indicating that C_60_-PDA-GSH nanoparticles indeed crossed the cell membrane and were more likely to accumulate in the perinuclear region of the cell. More specifically, the overlapped fluorescence signals of the labeled C_60_-PDA-GSH nanoparticles (green) and mitochondria-specific probes MitoTracker (deep red) implied C_60_-PDA-GSH nanoparticles were present in the mitochondria (Figure 8e–h), further demonstrating the internalization of C_60_-PDA-GSH nanoparticles after the incubation.

**Figure 9:**
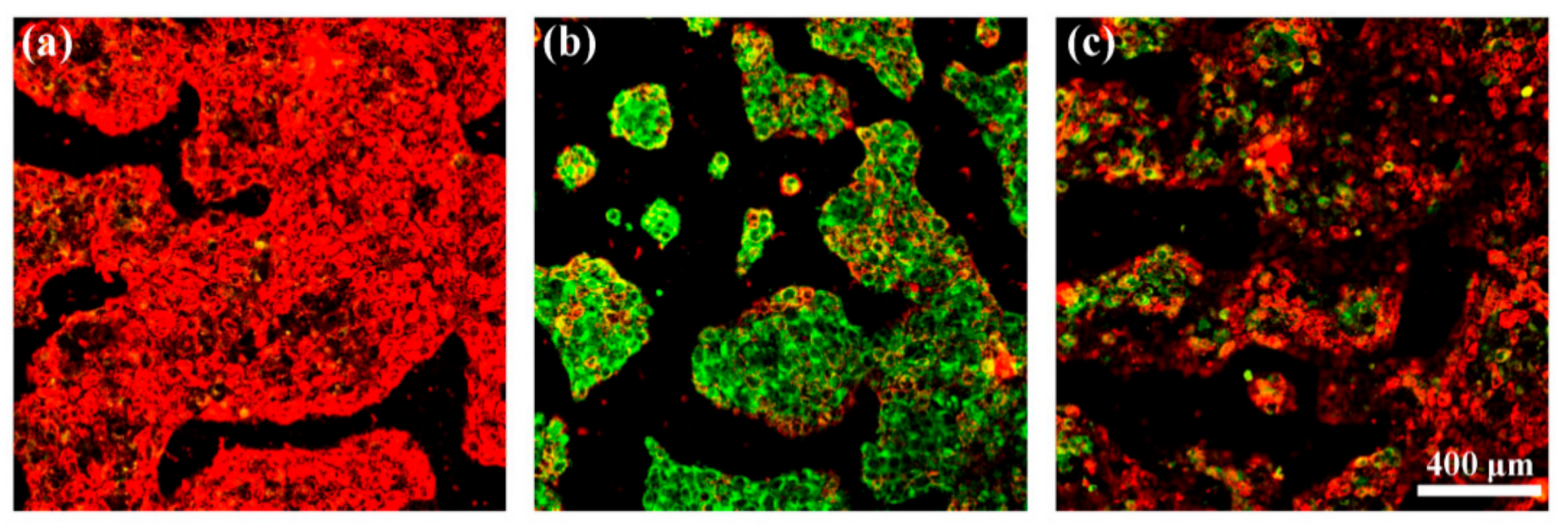
Confocal images with JC-1 assay of HEK-a cells incubated with (**a**) free culture medium as a control group, (**b**) PBS, and (**c**) 20 μg/mL C_60_-PDA-GSH nanoparticles for 3 h and then treated with 1 mM H_2_O_2_ for 1 h separately.

Considering the mitochondrial localization of C_60_-PDA-GSH nanoparticles as well as the cellular targets of ROS (including mitochondria), we employed a JC-1 probe regarding mitochondrial membrane potential (ΔΨ_m_) to assess their cytoprotective capacity against H_2_O_2_-induced mitochondrial damage in a visual mode. The transition of the JC-1 probe from red fluorescence to green fluorescence implies a drop in ΔΨ_m_ and an indicator of subsequent apoptosis. Figure 9 visually illustrated the cytoprotective properties of C_60_-PDA-GSH nanoparticles on HEK-a cells. For the control group without any addition, the JC-1 probe aggregated in cytoplasm to form a polymer (J-aggregates) and primarily produced red fluorescence, indicating the standard ΔΨ_m_ and healthy cells. Conversely, treatment with 1 mM H_2_O_2_ for 1 h triggered an apparent decrease in red fluorescence, and the green fluorescence generated by the JC-1 probe monomers increased significantly, suggesting the ΔΨ_m_ collapse and a severe oxidative injury to mitochondria. It is noteworthy that the green fluorescence intensity was indeed weakened when HEK-a cells were pretreated with 20 μg/mL C_60_-PDA-GSH nanoparticles compared to the group untreated, representing the ΔΨ_m_ loss being partially suppressed. This result is in accordance with the previous report that amino acid modified-gadofullerene inhibited cell apoptosis induced by oxidative stress [52]. Hence, our results from confocal microscopic images further manifested that C_60_-PDA-GSH nanoparticles could prevent oxidative damage to cell mitochondria, thereby stabilizing the mitochondrial membrane potential negatively affected by H_2_O_2_.

## 4. Conclusions

In summary, we have initially proposed a facile strategy for the large-scale preparation of functionalized fullerene-based nanoparticles, which was carried out gently without cumbersome procedures, external energy, catalysts, or organic solvents. The successful fabrication of C_60_-PDA-PEI nanoparticles via the combination of mussel inspired chemistry and Michael addition was confirmed by various characterization techniques. Compared with the pristine C_60_, the strong adhesion of PDA as well as the superior hydrophilicity of GSH enabled these C_60_ nanoparticles to achieve favorable water-solubility and biocompatibility. As a result, C_60_-PDA-GSH nanoparticles, acting as effective ROS scavengers, displayed a general protective role against oxidative stress on four kinds of human cells at concentrations as low as 2 μg/mL. Moreover, in light of the available attachment of abundant functional groups onto PDA-modified fullerenes, this easy-to-operate strategy would also provide insights and opportunities for potential applications of fullerene-based materials.

## Figures and Tables

**Table 1:** Element contents (%) of C_60_, C_60_-PDA, and C_60_-PDA-GSH based on the XPS analysis.

Samples	C1s (At%)	N1s (At%)	O1s (At%)	S2p (At%)
C_60_	97.50	0	2.50	0
C_60_-PDA	77.41	6.48	16.11	0
C_60_-PDA-GSH	66.51	9.08	22.14	2.27

## Supplementary Materials

The following are available online at https://www.mdpi.com/2079-4991/9/12/1647/s1, Figure S1: XPS C1s spectra and fitted curves of (**a**) C_60_-PDA and (**b**) C_60_-PDA-PEI nanoparticles; (**c**) S2p spectrum and fitted curve of C_60_-PDA-PEI nanoparticles, Figure S2: Zeta potential of C_60_-PDA-GSH nanoparticles measured by DLS.
