# Supplementary material for: Facile Synthesis of Water-Soluble Fullerene (C60) Nanoparticles via Mussel-Inspired Chemistry as Efficient Antioxidants
Source: Nanomaterials (Basel). 2019 Nov 20;9(12):1647. doi: 10.3390/nano9121647 (PMC6955807; doi:10.3390/nano9121647)
Supplement: Supplementary file 1 [file nanomaterials-09-01647-s001.pdf]

nanomaterials

MDPI

Article

Facile Synthesis of Water-Soluble Fullerene (C_{60}) Nanoparticles via Mussel-Inspired Chemistry as Efficient Antioxidants

Xiaoyan Zhang †, Yihan Ma *,†, Sheng Fu and Aiqing Zhang *

College of Chemistry and Materials Science, South-Central University for Nationalities, Wuhan 430074, China; zxy517317@163.com (X.Z.); fusheng4715@163.com (S.F.)

* Correspondence: mayihan@iccas.ac.cn (Y.M.); 074707@mail.scuec.edu.cn (A.Z.)

† These authors contributed equally to this work

Supporting Information

The characterization section:

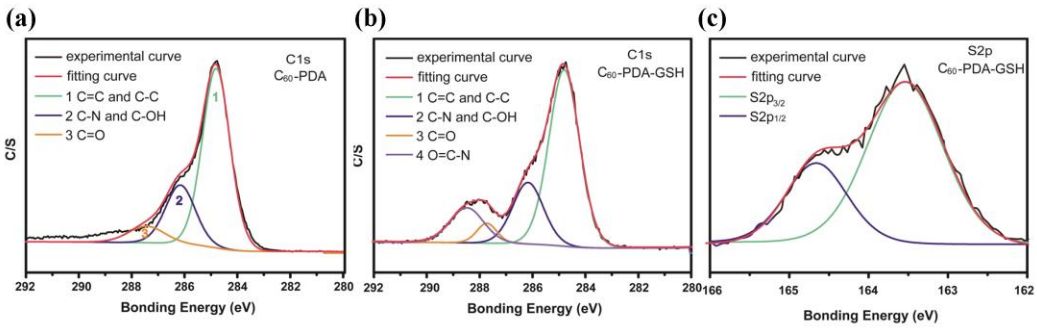

Figure S1. XPS C1s spectra and fitted curves of (**a**) C_{60}-PDA and **b**) C_{60}-PDA-PEI nanoparticles; (**c**) S2p spectrum and fitted curve of C_{60}-PDA-PEI nanoparticles

Dynamic Light Scattering (DLS) and zeta-potential measurements were performed on a Zetasizer Nano ZSP instrument. The samples were filtered through a membrane with 220 nm pore size before DLS measurement. The zeta potentials were measured three times per sample in Milli-Q water at pH 7.0.

nanomaterials

		Mean (mV)	Area (%)	St Dev (mV)
Zeta Potential (mV): -24.5	Peak 1:	-20.9	99.8	7.00
Zeta Deviation (mV): 24.1	Peak 2:	110	0.2	0.00
Conductivity (mS/cm): 0.0234	Peak 3:	0.00	0.0	0.00

Result quality : Good

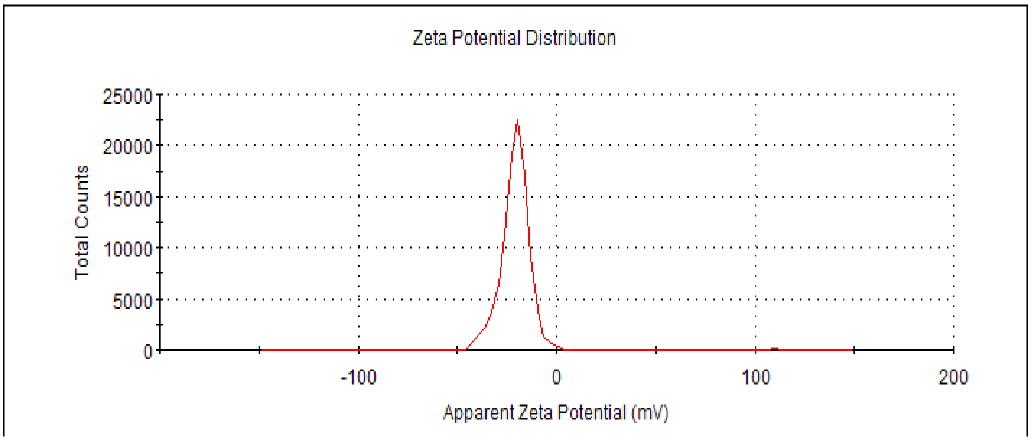

Figure S2. Zeta potential of C_{60}-PDA-GSH nanoparticles measured by DLS
